# Supplementary material for: Direct observation of coherent energy transfer in nonlinear micromechanical oscillators
Source: Nat Commun. 2017 May 26;8:15523. doi: 10.1038/ncomms15523 (PMC5458562; doi:10.1038/ncomms15523)
Supplement: Supplementary Information — Supplementary Figures, Supplementary Notes and Supplementary References [file ncomms15523-s1.pdf]

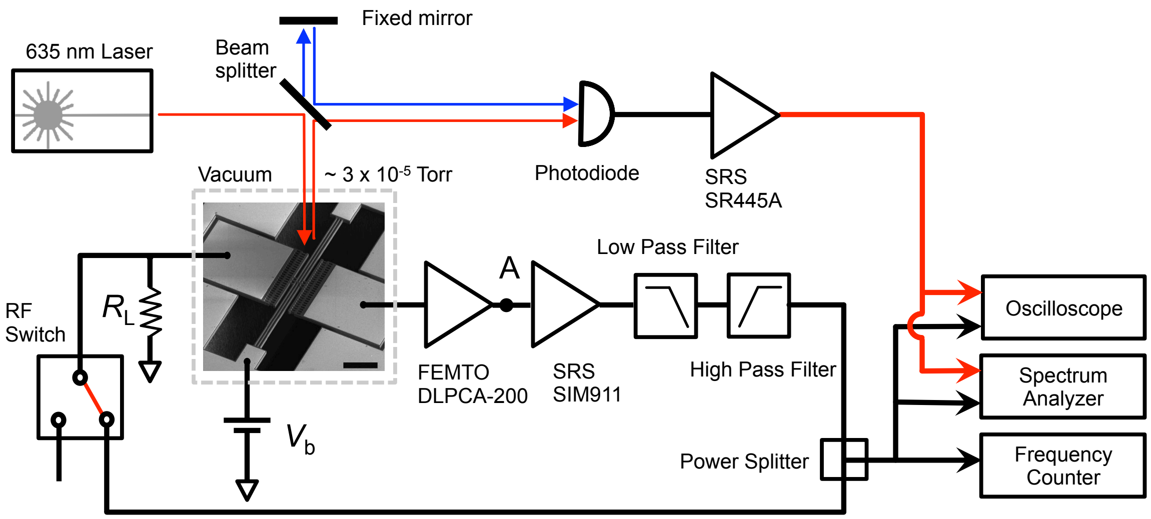

Supplementary Figure 1: Schematic of the complete setup with simultaneously electrical and optical interferometric measurements. When Switch 1 is set to position 2, we perform open-loop measurements to characterize the in-plane mode, and when both Switches 1 and 2 are set to position 1, we enable closed-loop configuration, either with or without a phase-locked loop (PLL), to allow self-sustaining vibrations. To trigger ringdown measurement, we turn Switch 2 from position 1 to position 2, and record the data with a digital oscilloscope.

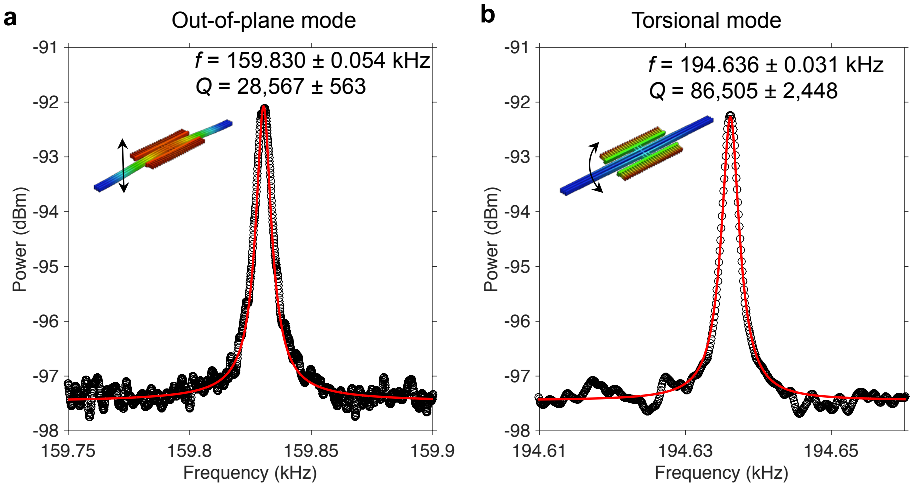

Supplementary Figure 2: Mechanical resonances driven by thermal noise at room temperature for **a**. the out-of-plane mode , and **b**. the torsional mode. The red lines are the Lorentzian fits to the data, with extracted resonant frequencies and quality factors shown. The uncertainties in the extracted values are due to functional fit processes. In our experiments, we did not observe IR interactions involving the out-of-plane mode.

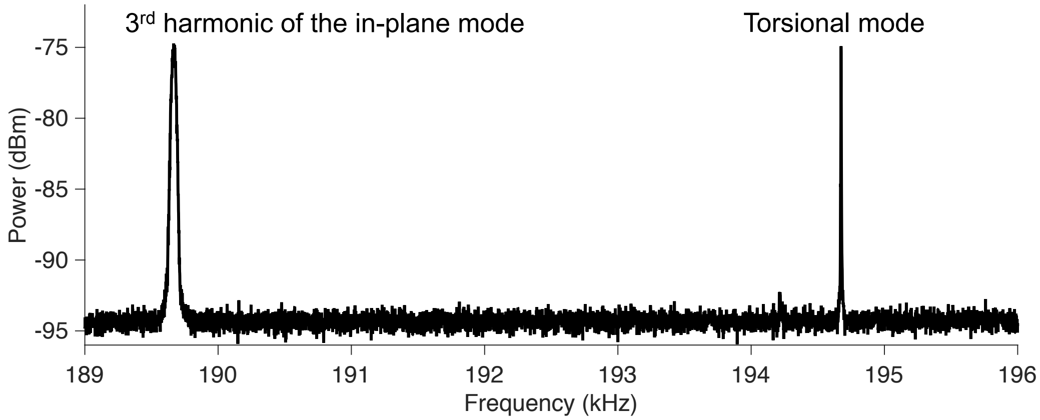

Supplementary Figure 3: Optical interferometric spectrum with in-plane mode is set to self oscillate at 63.2 kHz. Both the 3rd harmonic of the in-plane mode (189.6 kHz) and the torsional mode (194.6 kHz) are clearly visible. At internal resonance where the in-plane mode operates at 64.9 kHz, these two peaks overlap, preventing us from extract their individual amplitudes.

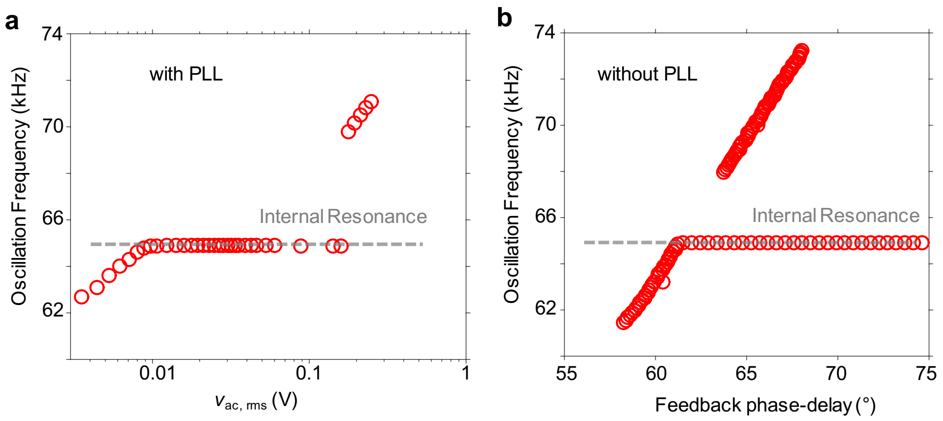

Supplementary Figure 4: Self-sustaining in-plane oscillation around IR approached with different configurations. a. With a PLL inserted in the feedback loop to control the excitation force ($\propto\ v_{ac}$), IR is approached, maintained, and exited by increasing v_{ac}. **b**. Without a PLL, the stability of the oscillation is maintained by the elastic nonlinearity of the c-c beam, and IR can be similarly approached, maintained and exited by adjusting the feedback phase-delay. The feedback phase-delay changes the in-phase excitation force. Note that for certain range of feedback phase-delay, there are multiple stable oscillations with different frequencies. The initial conditions of the oscillator will determine which of the stable oscillation is chosen, and these are achieved by sweeping the phase delay up and down through the hysteresis [1, 2].

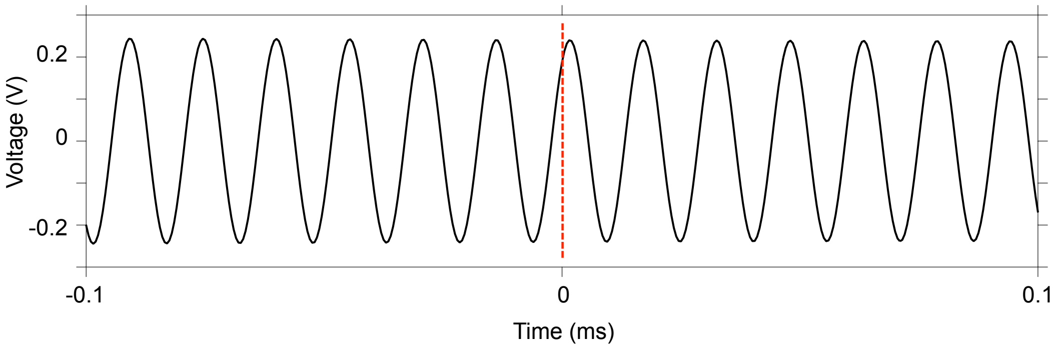

Supplementary Figure 5: Trigger profile. Zoomed in section around the trigger time at $t = 0$ s (red dashed vertical line), for Fig. 2b in the main text.

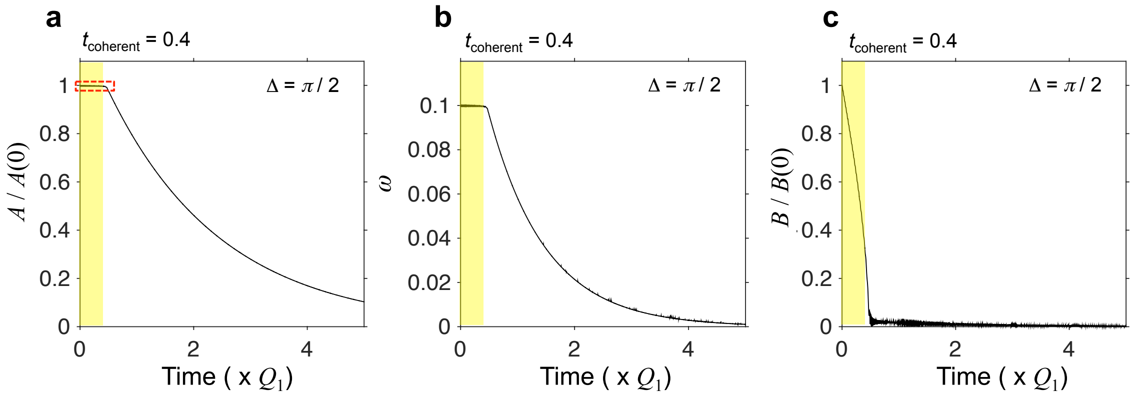

Supplementary Figure 6: Ring-down responses at IR. The phase delay $\Delta = \pi/2$, and the initial conditions are set at IR. **a**. Oscillation amplitude A of mode x_1, showing a coherent time of 0.4 (in scaled time unit). The area inside the red dashed box is shown in Supplementary Fig. 7. **b**. Oscillation frequency offset ω of mode x_1. **c**. Oscillation amplitude B of mode x_2, showing a fast decay during the coherent time. The system parameters used in the simulations are: $Q_1 = Q_2 = 10^5, \beta = 5 \times 10^{-3}, f_0 = 10^{-4}, \nu = 0.1, J = J' = 2 \times 10^{-4}$.

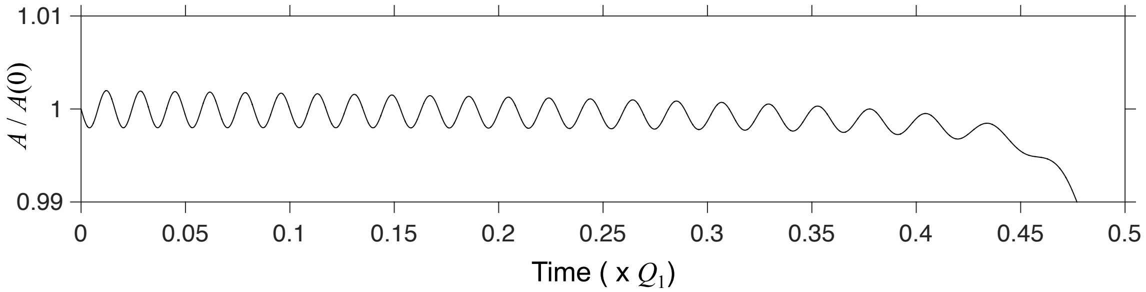

Supplementary Figure 7: Zoomed-in view of the oscillation amplitude $A(t)$ during t_{coherent}, from Supplementary Fig. 6**a**.

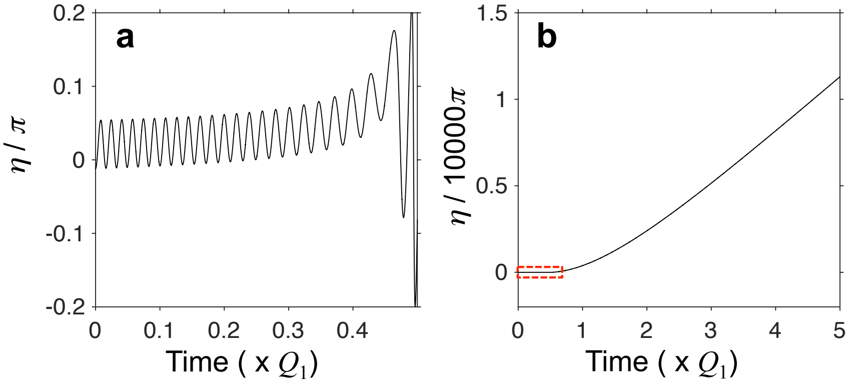

Supplementary Figure 8: Simulated phase difference. **a**. Simulated phase difference η during the beginning of ringdown. **b**. η over the whole simulated period. The red box indicates the portion shown in **a**.

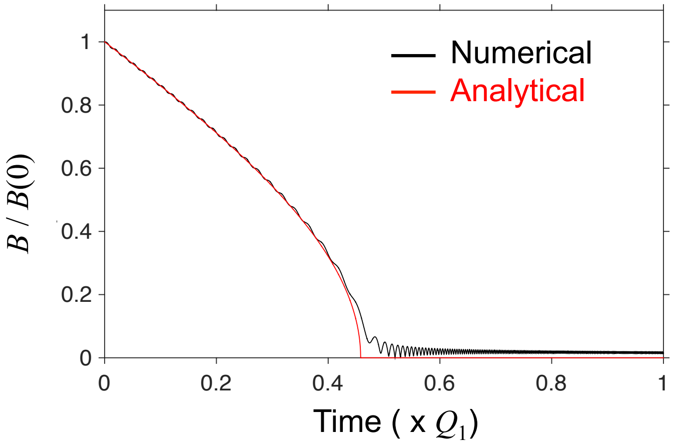

Supplementary Figure 9: Numerical and analytical solutions. Comparison between the numerical solution for B from eqs. 9 and analytical solution Supplementary Equation 12.

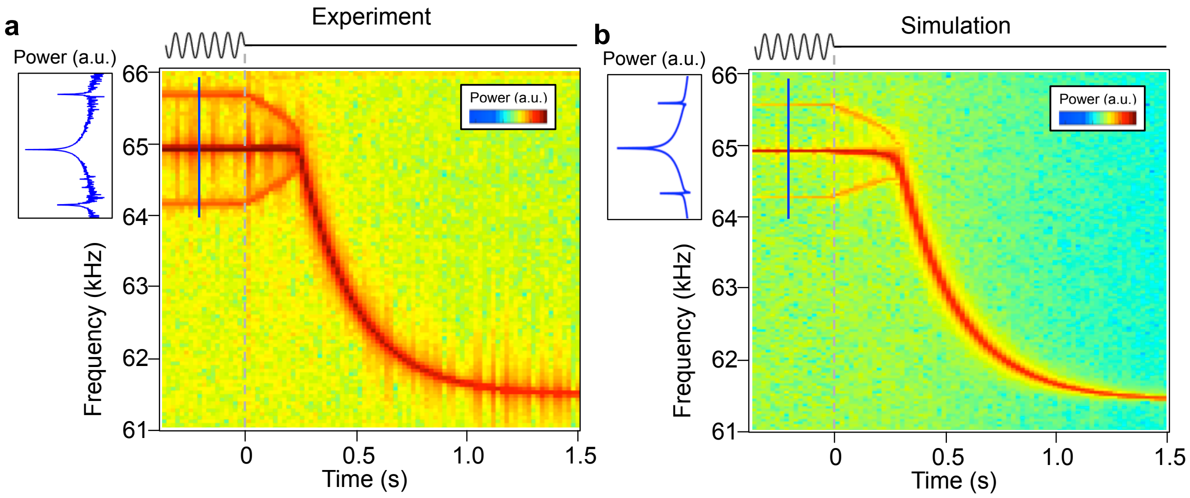

Supplementary Figure 10: Full numerical simulation. **a**. Temporal evolution of the instantaneous frequency at IR, before and after the external force is turned off at $t = 0$ s. This is the same fig. 4a in the main text. **b**. Same result obtained by numerically solving eqs. 8, with appropriately chosen parameters, and scaled back to the real time scale.

Supplementary Note 1: Analytical solution of steady-state motions for a single-mode nonlinear oscillator

In order to introduce the notation and approximations, we first analyze a single-mode, self-sustained Duffing oscillator, hence without the occurrence of IR. Then, we move on to discuss the case of two coupled modes, where IR arises.

We deal with the single-mode case as a generic one-dimensional oscillator whose departure from equilibrium is described by a coordinate $x_1(t)$ obeying [3]:

$$m_1\ddot{x}_1 + \gamma_1\dot{x}_1 + k_1 x_1 + \beta_1 x_1^3 = F_{\text{fb}}, \tag{1}$$

where $m_1, \gamma_1, k_1, \beta_1$ are the effective mass, damping rate, spring constant, and Duffing nonlinear constant, respectively, and F_{fb} is the external feedback force that drives the self-sustained oscillations. The force F_{fb} injects energy from external sources to replenish the intrinsic energy loss of the oscillator ($\gamma_1 > 0$), to maintain a stable oscillation amplitude. In the case of self-sustaining oscillator, the time-dependence of F_{fb} is determined solely by the system dynamics, without any additional frequency reference.

To simplify the notations, we define $\Omega_0 = \sqrt{k_1/m_1}$, $\epsilon = \gamma_1/m_1\Omega_0$, $\frac{4}{3}\beta = \beta_1/m_1\Omega_0^2$, and $f = F_{\text{fb}}/m_1\Omega_0^2$. Rescaling time such that the time unit is Ω_0^{-1}, Supplementary Equation 1 becomes:

$$\ddot{x}_1 + Q_1^{-1}\dot{x}_1 + x_1 + \frac{4}{3}\beta x_1^3 = f, \tag{2}$$

where $Q_1^{-1} = \epsilon$ is the quality factor. Assuming that, within the approximations considered below, the oscillations are well described by a harmonic function with time-dependent amplitude and phase, $x_1(t) = A(t)\cos\phi(t)$, the feedback force f must oscillate with the same frequency as x_1. Therefore, f can be expressed as

$$f = f_0 \cos[\phi(t) + \Delta], \tag{3}$$

where f_0 is the amplitude of the force, and Δ is the phase-delay induced by the feedback loop.

Given that $\epsilon \ll 1$, we can then apply the method of multiple time scales [4], which assumes that the oscillation period is much shorter than any other time scale related to the evolution of the amplitude and phase. This amounts to assuming that, in Supplementary Equation 2, $\ddot{x}_1$ and x_1 (which describe undamped, unforced, linear oscillations) are much larger than the other terms. Within this assumption, it is possible to isolate contributions from different time scales, leading to separate equations for the amplitude and frequency [1].

After expanding Supplementary Equation 2 and grouping the terms at different orders of ϵ, stationary self-sustained oscillations are obtained as a solution where the amplitude $A(t)$ and the frequency $\Omega_1(t) \equiv \dot{\phi}(t)$ are constant. The resulting equations are

$$-2\omega A + \beta A^3 = f_0 \cos\Delta \tag{4a}$$

$$Q_1^{-1} A = f_0 \sin\Delta \tag{4b}$$

where $\omega = \Omega_1 - 1$ is the detuning between the actual oscillation frequency and the natural frequency (normalized to 1). The Duffing resonance curve is obtained from these equations by fixing f_0 and solving for A and ω for different values of Δ [3, 5, 6]. In the limits of $Q_1^{-1}, f_0 \to 0$, we obtain the *backbone* approximation [1, 4]:

$$A = \sqrt{2\omega/\beta}. \tag{5}$$

Note that eqs. 4a and 4b can be interpreted as two "independent" equilibrium conditions. Supplementary Equation 4a describes the balance between elastic forces (both linear and nonlinear) and the in-phase component of the external force, whereas Supplementary Equation 4b describes the balance between the damping (Q_1^{-1}) and the out-of-phase component of the external force, which is maximal at the resonance peak $\Delta = \pi/2$. Near the resonance peak ($\cos\Delta \approx 0$), the system stability is essentially determined by the balance between the linear and nonlinear forces (left-hand side of Supplementary Equation 4a), while the external force is entirely involved in compensating the dissipation due to damping. This also means that if the quality factor is sufficiently large, only very small external force is needed to maintain the self-sustaining motion.

Supplementary Note 2: Ring-down response for single-mode nonlinear oscillator

After the system described by Supplementary Equation 2 has reached steady-state, hence oscillating with stable amplitude in the presence of the external feedback force, the ring-down process can be emulated by setting f to zero, as the external force is removed. We can still use the same approximations and method to obtain the time evolution for the amplitude and the phase of the oscillation. We now take $x_1(t) = A(t)\cos\phi(t)$ with $\phi(t) = t + \alpha(t)$, where $\alpha(t)$ represents the phase difference between the actual oscillation and the linear oscillation. Accordingly, the time-derivative $\dot{\alpha}(t)$ gives the instantaneous frequency difference between the actual oscillation and that of the linear frequency, which is normalized to 1. The multiple-scale approximation yields evolution equations for $A(t)$ and $\alpha(t)$:

$$\dot{A} = -\frac{1}{2}A/Q_1, \tag{6a}$$

$$\dot{\alpha} = \frac{1}{2}\beta A^2, \tag{6b}$$

whose solutions are:

$$A(t) = A_0 \exp(-t/2Q_1), \tag{7a}$$

$$\alpha(t) = \alpha_0 + \frac{1}{2}\beta Q_1 A_0^2 \left[1 - \exp(-t/Q_1)\right], \tag{7b}$$

with A_0 and α_0 the initial conditions obtained from the steady-state solutions.

We see that the amplitude $A(t)$ decays exponentially towards zero, with a time constant of $2Q_1$, while the phase $\alpha(t)$ decays exponentially with a time constant of Q_1, i.e., twice as fast as $A(t)$. The instantaneous frequency, $\dot{\phi}(t) = 1 + \dot{\alpha}(t)$, decays exponentially from its initial value $1 + \omega$ towards 1 with the same time constant of Q_1. Moreover, according to Supplementary Equation 6b, the instantaneous frequency difference $\dot{\alpha}$ is related to the instantaneous amplitude A exactly as the steady-state frequency ω and steady-state amplitude A along the backbone curve, Supplementary Equation 5. Therefore, the decay of the amplitude and the frequency during ring-down should take place along the backbone curve.

Supplementary Note 3: Steady-state solution for the coupled-mode motion and emergence of internal resonance (IR)

We model IR by coupling the above Duffing oscillator (main mode) with a linear oscillator representing a higher frequency vibrational mode. We disregard the factor 3 in the frequency of the higher mode with respect to that of the main mode and, therefore, assume that the linear frequencies of the two modes are close to each other. With these assumptions, the coupled-mode system is described by:

$$\ddot{x}_1 + Q_1^{-1}\dot{x}_1 + x_1 + \frac{4}{3}\beta x_1^3 = f_0\cos(\phi + \Delta) + Jx_2, \tag{8a}$$

$$\ddot{x}_2 + Q_2^{-1}\dot{x}_2 + (1+\nu)^2 x_2 = J' x_1. \tag{8b}$$

Here, Q_2 and $(1+\nu)$ are the quality factor and the natural frequency for mode x_2, respectively, and J and J' denote the coupling strengths between the modes.

As in the preceding section, we assume the motions of mode x_1 takes the form of $x_1(t) = A(t)\cos[t + \alpha(t)]$, and now, mode x_2 takes the form of $x_2(t) = B(t)\cos[t + \zeta(t)]$. According to our assumptions, the amplitudes A, B and the phases difference α and ζ evolve slowly as compared with the oscillations. Therefore, after plugging the proposed solutions for mode x_1 and x_2 into eqs. 8, and neglecting small time derivative terms, we have:

$$2\dot{A} = -Q_1^{-1}A + f_0\sin\Delta + JB\sin\eta, \tag{9a}$$

$$2A\dot{\alpha} = \beta A^3 - f_0\cos\Delta - JB\cos\eta, \tag{9b}$$

$$2\dot{B} = -Q_2^{-1}B - J'A\sin\eta, \tag{9c}$$

$$2B\dot{\zeta} = 2\nu B - J'A\cos\eta, \tag{9d}$$

with $\eta = \zeta - \alpha$ is the phase difference between the two modes. As expected, the case considered in the preceding sections are obtained for $J = J' = 0$.

Internal resonance is described by the steady-state solutions to eqs. 9 when both modes have stable oscillations ($\dot{A} = \dot{B} = 0$) with non-zero amplitudes ($A, B \neq 0$), and are mutually synchronized with the same frequency of $\Omega = 1 + \omega$, so that $\dot{\alpha} = \dot{\zeta} = \omega$. The details of the steady-state solutions can be found in Refs. [1, 2]. Note that, with given system and control parameters, there might be multiple stable solutions, as well as complicated sets of instabilities and bifurcations. Internal resonance corresponds to the particular solution in which $\omega < \nu$, and this steady-state solution will be used as the initial conditions for the following transient simulation.

Supplementary Note 4: Ring-down response for the coupled-mode system at IR and derivation of t_{coherent}

Given the steady-state solutions as initial conditions, the same governing equations, eqs. 9 can be readily solved numerically to obtain the ring-down response at IR, by setting $f_0 = 0$. Supplementary Fig. 6 shows the simulated ring-down for both modes, with the system parameters shown in the caption. We can clearly see the nearly constant oscillation amplitude and frequency for mode x_1, until a time about 0.4 (in scaled unit). During the same period of time, the oscillation amplitude of mode x_2 drops rapidly and non-exponentially. As mode x_2 reaches a very low amplitude, mode x_1 (both amplitude and frequency) begins its decay.

However, if we zoom in to the oscillation amplitude $A(t)$ during t_{coherent}, we see that $A(t)$ oscillates around its initial value, with a very small, slowly decaying amplitude, and a slowly decreasing frequency. The origin of such fine structure can be explained as follows: when the external force is turned off, the forces that maintained the stationary oscillation become unbalanced. This unbalance, however, is rather slight since the oscillator has large quality factor, and the self-sustaining force necessary to maintain the oscillation is very small as compared with other involved forces (i.e., linear and cubic elastic forces, see Supplementary Equation 4). At the same time, the external action that maintained the synchronization between the two modes disappears, and the two modes will behave as two coupled, weakly damped oscillator with different natural frequencies and no mechanism to sustain their synchrony and to counteract the energy dissipation. However, the main mode (mode x_1) has not left far from its previous force-balanced state, therefore it will take some time for the main mode to move away.

The immediate consequence of the desynchronization between the two modes is that their phases, whose difference η was previously constant, begin to oscillate with respect to each other. This is also seen in the numerical simulation (Supplementary Fig. 8). The oscillation of the relative phase η, in turn, implies an oscillating flow of energy between the two modes: a negative η implies energy flow from mode x_1 to mode x_2, and vice versa. The oscillation of η explains the oscillation in A, corresponding to the energy exchange.

Furthermore the simulated transient responses of η shows that it oscillates around a positive value, which implies that, despite of oscillation of the direction of energy flow, there is a net energy flow from mode x_2 to mode x_1. Consequently, the amplitude B of mode x_2 decays rather abruptly, while the amplitude A of mode x_1 remains practically constant. This pseudo-equilibrium, however, breaks down as soon as mode x_2 exhausts its energy. From then on, mode x_1 moves as in the absence of the other mode, i.e., as a single-mode nonlinear oscillator, and decays along the backbone curve as described in previous section.

In order to gain more physical insight beyond the numerical results, especially a compact expression of t_{coherent}, we next attempt to obtain an analytical solution to eqs. 9 during ring-down ($f_0 = 0$). Based on the numerical results, we make the following further assumptions: (i) the phase difference η remains small, during the early portion of the ring-down; (ii) in eqs. 9b and 9d, the dominant terms are those corresponding to the internal dynamics of each mode, while those related to their interaction are relatively small; (iii) the value of A remains close to its initial value, which can be approximated by the position of the IR gap in the resonance curve as $A_{\text{IR}} = \sqrt{2\nu/\beta}$; and (iv) the oscillations of mode x_1 and mode x_2 are much faster than the decay of B. With these assumptions, we can rewrite eqs. 9 as:

$$2\dot{A} = -Q_1^{-1}A + JB\eta, \tag{10a}$$
$$2A\omega = \beta A^3 - JB, \tag{10b}$$
$$2\dot{B} = -Q_2^{-1}B - J'A\eta, \tag{10c}$$
$$2B\omega = 2\nu B - J'A. \tag{10d}$$

Since t_{coherent} is directly connected to the decay of B, we will focus our effort on Supplementary Equation 10c. Given the assumption of $A_{\text{IR}} = \sqrt{2\nu/\beta}$ at $t = 0$, and plugging it to Supplementary Equation 10a, we obtain $\eta =$

$A_{\mathrm{IR}}/(BQ_1J)$, therefore, Supplementary Equation 10c becomes:

$$2\dot{B} = -\frac{1}{Q_2}B - \frac{J'}{J}\frac{2\nu}{\beta Q_1}\frac{1}{B}, \tag{11}$$

which then can be solved analytically. The solution is given as:

$$B(t) = \left(\frac{\exp(\mathbb{C}+at)-b}{a}\right)^{1/2}, \tag{12}$$

where $a = -Q_2^{-1}$, $b = -\frac{J'}{J}\frac{2\nu}{\beta Q_1}$, and $\mathbb{C}$ is a constant determined by the initial conditions such that $B(0) = (\frac{\exp(\mathbb{C})-b}{a})^{1/2}$. Supplementary Fig. 9 shows that the analytical solution (Supplementary Equation 12) shows good agreement to the numerical result.

Our interest is then to find the time where $B(t)$ becomes zero, and identify this value as t_{coherent}:

$$t_{\mathrm{coherent}} = Q_2 \ln\left(\frac{a}{b}B^2(0)+1\right). \tag{13}$$

The only remaining unknown is $B(0)$, which is the steady-state oscillation amplitude at IR for mode x_2. For this we retrieve the identity relation of:

$$\sin\eta = -\frac{B(0)}{Q_2J'A_{\mathrm{IR}}}. \tag{14}$$

Plugging Supplementary Equation 14 back to equations at steady-state, and after some algebra, we have:

$$B^2(0) = A_{\mathrm{IR}}Q_2\left(-\frac{A_{\mathrm{IR}}}{Q_1}+f_0\sin\Delta\right)\frac{J'}{J}, \tag{15}$$

and finally:

$$t_{\mathrm{coherent}} = Q_2^{-1}\ln\left(\frac{Q_1(f_0\sin\Delta)}{A_{\mathrm{IR}}}\right), \tag{16}$$

with $A_{\mathrm{IR}} = \sqrt{2\nu/\beta}$, and this is the form that we used to fit the t_{coherent} shown in Fig. 2e.

Supplementary Note 5: Full numerical simulation

The solutions for eqs. 8 with the perturbation method outlined above are aimed to obtain the dynamics of the vibrational amplitudes $A(t)$, $B(t)$, and the primary frequency component $\omega(t)$, but not the full evolution of $x_1(t)$ and $x_2(t)$, which may consist of multiple frequency components that arises from the coupling. In order to reveal the full dynamics of the coupled system, we used numerical method (`scipy` module from `Python`) to brutal-force numerically solve for the time-domain solution of eqs. 8, with appropriately chosen system parameters. The comparison between the experimental data and full numerical simulation is shown in Supplementary Fig. 10. The time unit of simulation result is scaled back to the real time scale.

Supplementary Note 6: Conversion between linear coupled model and linearly uncoupled normal form model

Note that if x_1 and x_2 represent a pure flexural and torsional degree of freedom (DOF), respectively, the coupling in the x_j's results from some asymmetry in the mechanical and/or electrostatic restoring force on those DOFs (as described in previous section). Then the eigen-modes of the system, expressed by coordinates q_1 and q_2, are not pure flexure and pure torsion, but some perturbations thereof. The equations for the q_j's are necessarily uncoupled at linear

order. The eigen-modes of this system are distinct from, but close to, the x_j in this case. Since the flexure DOF (x_1) is measured in the experiments, we focus on the x_j model in the main text. However the essential coupling is more naturally described by the q_j model, as developed here.

Starting with the linear coupled model for the x_j's, i.e., equation (1) in the manuscript, we scale the second equation so that the coupling coefficients (cross-stiffnesses) are equal, hence making the problem self-adjoint:

$$\ddot{x}_1 + \gamma_1 \dot{x}_1 + x_1 + \gamma x_1^3 = J x_2, \tag{17a}$$

$$\frac{J}{J'} \left(\ddot{x}_2 + \gamma_2 \dot{x}_1 + \Omega_2^2 x_2 \right) = J x_1, \tag{17b}$$

where $\gamma = \frac{4}{3}\beta$. For this development we can ignore damping, hence setting $\gamma_i = 0$, and we will consider the case of 3:1 resonance ($\Omega_2 \approx 3$). We assume the solutions in the form of $x_i = X_i \cos(\omega t)$, then the linear governing equation can be expressed in matrix form as:

$$\begin{pmatrix} 1-\omega^2, & -J \\ -J, & \frac{J(\Omega_2^2-\omega^2)}{J'} \end{pmatrix} \begin{pmatrix} X_1 \\ X_2 \end{pmatrix} = 0. \tag{18}$$

Setting the determinant of the above matrix to zero, we can solve for the eigen-frequencies, $\omega = \omega_{1,2}$ of the system. If we assume that $\Omega_2 = 3(1+\nu)$, where $|\nu| \ll 1$, we can obtain:

$$\omega_1 = \sqrt{5 - \Delta + 9\nu(1 - 4\Delta^{-1})} \approx 1 - \frac{1}{16} J J', \tag{19a}$$

$$\omega_2 = \sqrt{5 + \Delta + 9\nu(1 + 4\Delta^{-1})} \approx 3\left(1 + \nu + \frac{1}{144} J J'\right), \tag{19b}$$

where $\Delta = \sqrt{16 + JJ'}$ and the approximations on the right use expansions in both detuning, $|\nu| \ll 1$, and coupling, $|J|, |J'| \ll 1$. As seen from Supplementary Equation 19, the linear coupling coefficients (J, J') provides an additional frequency detuning away from the 1:3 condition between the uncoupled modes. This coupling and the Duffing nonlinearity combine to provide the coupling between eigen-modes, as shown below. However, the detuning parameter ν will not affect the ultimate nonlinear coupling terms at leading order.

The conversion of the governing equations from x_j to q_j is acheived by a standard similarity transformation using the system eigen-modes, u_j, and carrying along the nonlinear terms in the transformation. The eigen-modes, normalized by the inertia terms, are given by:

$$u_j = \mu_j^{-1} \left(1\,,\ \frac{1-\omega_j^2}{J}\right)^T, \tag{20}$$

where $\mu_1 = \sqrt{\frac{2\Delta(\Delta-4)}{JJ'}}$ and $\mu_2 = \sqrt{\frac{2\Delta(\Delta+4)}{JJ'}}$. We form the transformation matrix U with the u_j's as columns and define new variables, the eigen-coordinates q_i, using $(x_1, x_2)^T = U(q_1, q_2)^T$. The transformation matrix is given by

$$U = \begin{pmatrix} \mu_1^{-1} & \mu_2^{-1} \\ \frac{1-\omega_1^2}{J\mu_1} & \frac{1-\omega_2^2}{J\mu_2} \end{pmatrix} \approx \begin{pmatrix} 1 - \frac{JJ'}{128} & \frac{\sqrt{JJ'}}{8} \\ \frac{JJ'}{8} & -\sqrt{\frac{J'}{J}}\left(1 - \frac{JJ'}{128}\right) \end{pmatrix} \tag{21}$$

where the approximations on the right are obtained by expanding in ν, J, and J'. Transforming the equations of motion, Supplementary Equation 17 (without dissipation), to modal coordinates q_i, and using the expansions in ν, J, and J' yields the following normal form model:

$$\ddot{q}_1 + \omega_1^2 q_1 + \gamma q_1^3 + \frac{3}{8}\gamma\sqrt{JJ'} q_1^2 q_2 = 0, \tag{22a}$$

$$\ddot{q}_2 + \omega_2^2 q_2 + \frac{1}{8}\gamma\sqrt{JJ'} q_1^3 = 0. \tag{22b}$$

Note that only resonant nonlinear terms survive the transformation and expansion, and these can be derived from a nonlinear coupling potential $H_{12} = \frac{1}{8}\gamma\sqrt{JJ'} q_1^3 q_2$. Also note that the Duffing nonlinearity acts only on the first mode,

This formulation confirms that linear coupling combined with a Duffing nonlinearity in the x_j (DOF) model leads to a nonlinear coupling in the q_j (modal) model, and one can directly compare the two descriptions of coupling. A key point is that the coupling and Duffing nonlinearity in the DOF allows nonlinear resonant energy exchange between the eigen-modes.

Supplementary References

[1] S. I. Arroyo and D. H. Zanette, The European Physical Journal B **89**, 1 (2016).

[2] F. Mangussi and D. H. Zanette, PloS one **11**, e0162365 (2016).

[3] R. Lifshitz and M. C. Cross, in *Reviews of Nonlinear Dynamics and Complexity* (Wiley-VCH Verlag GmbH & Co. KGaA, 2009) pp. 1–52.

[4] A. H. Nayfeh and D. T. Mook, *Nonlinear oscillations* (John Wiley & Sons, 2008).

[5] L. G. Villanueva, R. B. Karabalin, M. H. Matheny, E. Kenig, M. C. Cross, and M. L. Roukes, Nano Lett. **11**, 5054 (2011).

[6] C. Chen, D. H. Zanette, J. R. Guest, D. A. Czaplewski, and D. López, Physical Review Letters **117**, 017203 (2016).
